# Identification of potential hydropower generation sites using geospatial techniques in the Megecha watershed of Ethiopia

**DOI:** 10.1016/j.heliyon.2025.e42063

**Published:** 2025-01-21

**Authors:** Habtamu Wimego Anore, Tarun Kumar Lohani, Abebe Temesgen Ayalew

**Affiliations:** Faculty of Hydraulic and Water Resources Engineering, Water Technology Institute, Arba Minch University, P.O. Box 21, Arba Minch, Ethiopia

**Keywords:** Geospatial analysis, Multicriteria decision-making, Hydropower potential modelling, Megecha watershed, Ethiopia

## Abstract

The research focuses on modelling the surface water potential using Arc SWAT and Multi-criteria decision-making (MCDM) for selecting the potential hydropower sites in the Megecha watershed. The tenure for stream flow and meteorological data used for SWAT model simulation is from 1990 to 2019 and 1988–2019 respectively. Geospatial method is used to generate gross head and respective potential hydropower. Multi criteria decision method is adopted to select the best site for hydropower generation. Curve number (CN2), saturated hydraulic conductivity (SOL_K), moist bulk density (SOL_BD), and available moisture in the soil layer (SOL_AWC) were among the most sensitive parameters assessed during the research. The performance of SWAT model obtained for R^2^, NSE and PBIAS were 0.84, 0.78 and −4.6 and 0.81, 0.72 and −4.8 in percentages during calibration and validation periods, respectively. Based on the flow duration curve, the minimum flow of the river for the percent of exceedance of 90 % (Q_90_) for the sites 1, 2, and 3 are 0.37 m^3^/s, 0.45 m^3^/s, and 0.48 m^3^/s, respectively. The available heads and corresponding hydropower potential on the identified sites were 15, 25, and 20 m and 54.45 KW, 110.36 KW, and 94.18 KW at site1, 2, and 3, respectively. From the GIS based MCDM analysis, the best alternative site for hydropower generation is at site 2, in comparison to identified sites. The total watershed area was 1176 km^2^ of which, 308.65 million m^3^ of runoff was generated by the model annually. The watershed has high surface water potential, and the rivers have enough surface water that may be used for hydropower development.

## 1 Introduction

Surface water is a finite renewable resource available on the surface of the Earth [1,2], whose quantity and quality are both space- and time-dependent. Lakes and rivers that serve as the major sources of water constitute less than 0.3 % out of nearly 3 % of the Earth's freshwater [3,4]. However, the availability of freshwater in many regions is likely to decrease due to population growth, industrialization, land use, and climate change [5]. Therefore, estimating the surface water potential of a river basin is crucial for the future development of any water-related projects. Ethiopia has huge surface water potential for hydropower generation and the estimated hydropower potential of the Ethiopia is around 45,000 MW but still the current hydropower generation capacity is nearly 10,000 MW after the completion of Grand Ethiopian Millennium Dam [6,7]. Surface water flow in river basin systems is amazing as almost all river basins are formed in highland regions with abundant rainfall. Because of the water sector's poor administration, technical difficulties, and inadequate financial resources, this potential is not judiciously utilized and transformed into development. Surface water resources in this region are mainly the result of overland runoff of rainwater and in some regions, they also originate from the groundwater in the form of springs. These surface water resources are the primary sources to generate hydropower.

This study was conducted to assess the surface water potential of the Megecha watershed lying in the northeast part of the Omo-Gibe basin for selecting potential sites to estimate the hydropower potential of the watershed. An attempt was undertaken to assess the surface water potential of the watershed to choose the potential sites in the watershed, estimate the flow of the river, and provide renewable power to the community.

Hydropower is one of the most promising types of renewable energy sources [4,8]. Probably, it is the most cutting-edge technology used to convert natural water energy to electrical energy using turbines, when compared to other sources of renewable energy [9,10]. The source of this energy is the natural flow of water in a river. To generate this power from the streamflow, it is essential to evaluate and estimate the water potential of the river basins or catchments. In terms of intergenerational equity, hydropower has a major contribution to produce one of the main sustainable development aspects, since a significant part of the production costs comes at the construction stage, though the projects have a prolonged existence [11,12]. Once constructed and capital expenditure is estimated, a project is virtually immune to further inflationary pressures.

Ethiopia is the richest country in water resources in Africa from which ample quantity of hydropower can be generated [4,[13], [14], [15]]. Unfortunately, 90 % of the energy consumed in the country is derived from biomass fuels. And around 8 % is energy in the country is consumed from water even the country has huge potential for hydropower potential [16]. The use of biomass as the source of energy has resulted in massive deforestation and subsequent soil erosion. The existing situation indicates that the supply and demand of power are not balanced, and the demand is much higher than the supply.

Regression analysis and rainfall-runoff models are used in a limited but expanding body of research to estimate the amount of energy produced by a hydroelectric system subject to weather-induced changes in river flow [6]. One way around the dearth of large-scale, reliable data is to use rainfall-runoff models in combination with remote-sensing datasets. In the meanwhile, the researcher can determine the connection between water availability and electricity generation by regression analysis. Using the GeoSFM model and regression techniques [17], evaluate the possible hazards to energy supply in hydro-dependent African nations under several IPPC climate change scenarios. This is one of their notable articles. Two studies are available in this studies for hydropower generation [12], conducted an integrated research using the CLIRUN-II model to simulate river flow, and [18] assessed the effects of climate change on several water-dependent sectors, including the energy sector in Vietnam.

The Megecha watershed in the Omo-Gibe basin has a huge hydropower potential with temporal and geographical variations being evaluated via the use of the SWAT hydrological model. The SWAT model was used to evaluate the head, choose sites, and simulate flow at each chosen location [[19], [20], [21]]. Each stream inside the watershed boundary's length, elevation difference, and stream network properties are produced using the SWAT model. The model allows users to add or remove outlets and inlets, which changes the number and delineation of sub-watersheds the model creates. This feature was used to measure head variation throughout the river by positioning sub-basin outlets at different locations. Geographic Information System (GIS) and Soil and Water Assessment Tool (SWAT) models have been shown in recent research to be the most widely used and economically viable methods for estimating river discharge and detecting potential hydropower [19,22,23].

In studying the surface water potential in river catchments to estimate hydropower, hydro-meteorological issues remain the main concern [[24], [25], [26]]. Previously, hydropower plant developers neither carry out their projects considering the specific objectives nor taking any scientific methods discussing with the concerned bodies in the government institutions or the social representatives. It did not generate any concern until the shortage of flow was anticipated and caused tremendous hazards to the downstream ecosystems raising social conflicts. Currently, it has been the common and vital practice to carefully investigate and estimate the hydro-meteorological issues on the specified catchment or watersheds before any other investigations are undertaken to execute water resource projects. The Megecha watershed surface water assessment is conducted to estimate the surface water potential and to select prospective sites for hydropower estimation on the river, especially in the Southern Nations, Nationalities, and Peoples’ Region (SNNPR) and in the Gurage zone of Omo Gibe River basin.

## 2 Materials and methods

### 2.1 Description of the study area

**Fig. 1:**
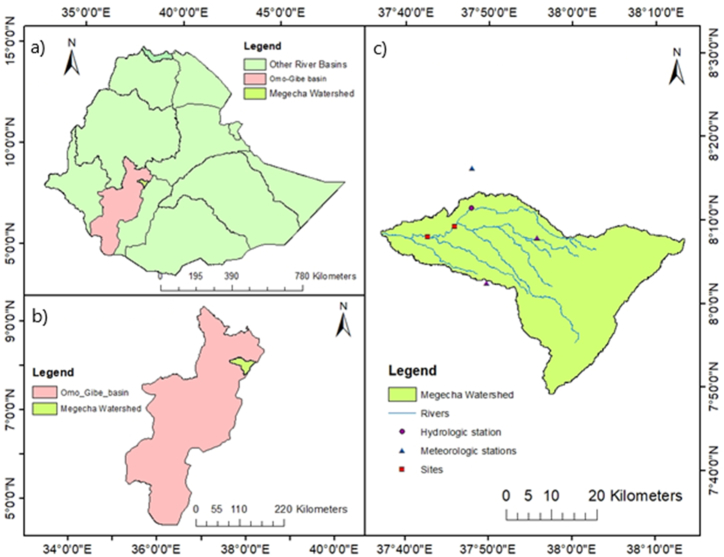
a) Ethiopian River Basins; b) Omo-Gibe Basin; c) Megecha Watershed.

The Megecha watershed is one of the major tributaries of the Omo-Gibe River basin originated in the northeast of the basin (Fig. 1a). The Megecha River, which flows from west to southwest across south central Ethiopia, is bounded by SNNPR's Gurage Zone (Fig. 1b). Geographically, the study area is situated between 7°49′12″ to 8°13′30″N Latitude and 37°36′21.6″ to 38°12′39.6″E Longitude, covering an area of about 1176 km^2^ (Fig. 1c).

#### 2.1.1 Topography and slope

**Fig. 2:**
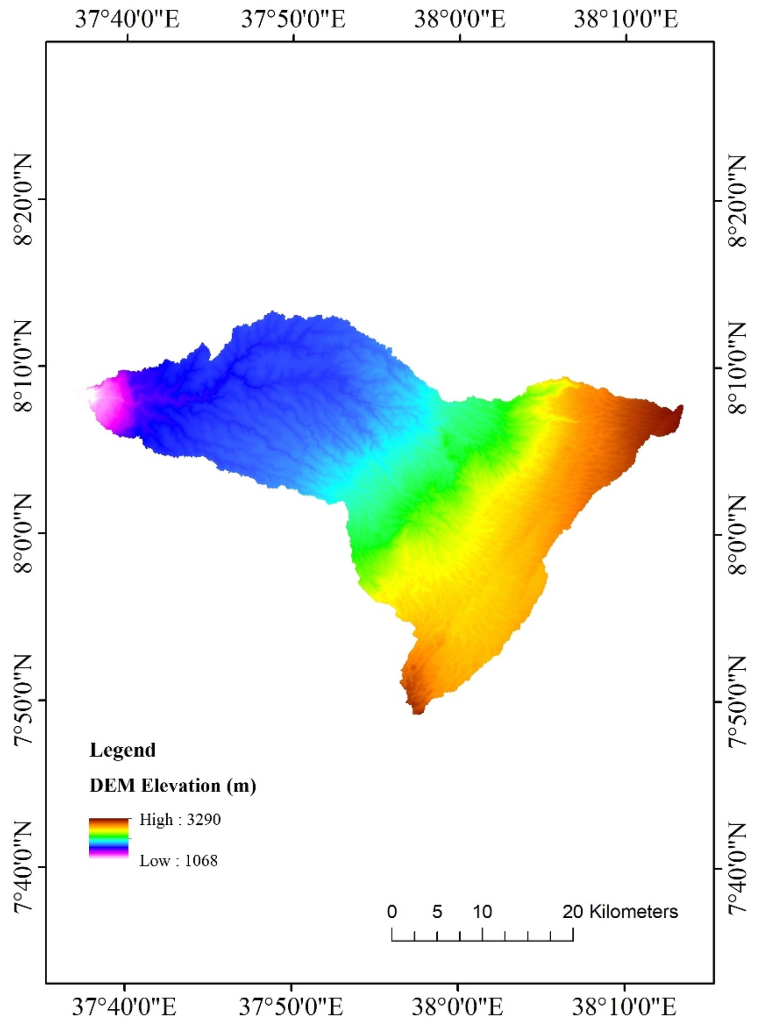
The DEM map of the Megecha watershed.

The Megecha watershed is characterized by rugged land separated by tributaries in its eastern part and relatively flat lands in the western part until the outlet is reached. The river flows from the higher elevation of 3290m to a minimum altitude of 1050m with an average slope of 11.32 %. In this study, 12.5 × 12.5m resolution DEM data derived from the Alaskan satellite facility website was used to estimate slopes and other physiographical characteristics of the watershed (Fig. 2).

#### 2.1.2 Land use/land cover

**Fig. 3:**
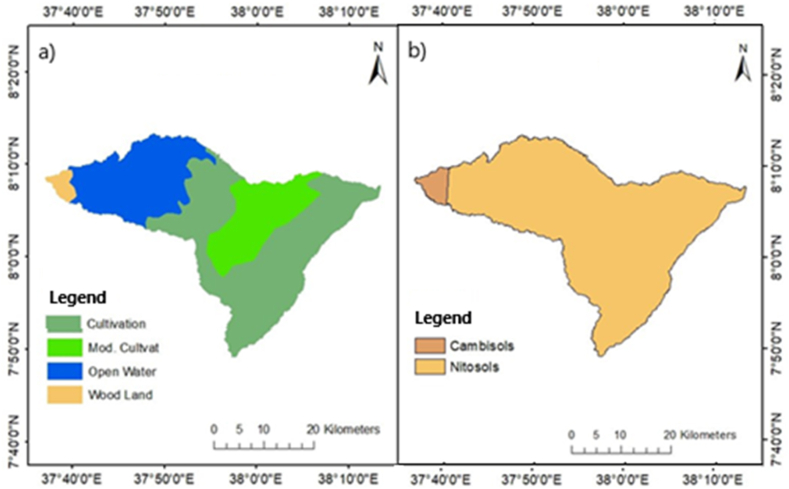
a) Land use/land cover; b) Soil types in the Megecha watershed.

The land use pattern of the study area is characterized by extensive agricultural lands and some parts are used for pastoral purposes. According to the field visit that was conducted during the collection of some available data; around 10 % of forest areas are now confined to cultivation and open water lands only (Fig. 3a).

#### 2.1.3 Soil types

Red and reddish-brown clay looms over clays in the majority of the deep to extremely deep soils were found across the Omo-gibe River basin. These soils are well-drained and are widespread over the entire northern basin. Since, the Megecha watershed is one of the northeastern catchments of the basin, it is mostly covered by clay soil (Nitosols) and a minor part of the watershed is covered by loam soil (Cambisols) (Fig. 3b).

#### 2.1.4 Climate

**Fig. 4:**
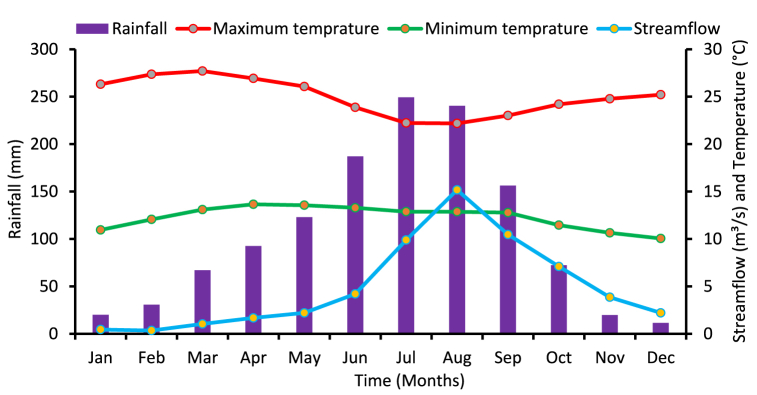
Mean monthly precipitation, temperature, and streamflow of Megecha River.

Megecha watershed is one of the main tributaries of the Omo Gibe river basin existing in the northeastern part, characterized by a tropical sub-humid climate. From May to September the intensity of rain is high. Nonetheless, this season's wettest months are July and August. Average annual rainfall ranges from nearly 774 mm in minimum to a maximum of 1600 mm. The typical ranges for maximum and lowest temperatures are 22.21 °C–27.71 °C and 9.72 °C–13.47 °C, respectively. March has the highest temperature (27.7 °C) (Fig. 4).

#### 2.1.5 Stream flow

The primary objective of the hydrological study is to simulate stream flow, since

stream flow is the key variable to determine the potential water resource availability. In addition, it is also the main parameter to estimate the theoretical hydropower potential of the river for any proposed purposes. The steam flow data of the watershed is gauged near Gubrie and the mean monthly stream flow from the year 1990–2006 is presented in Fig. 4.

### 2.2 Methods

**Fig. 5:**
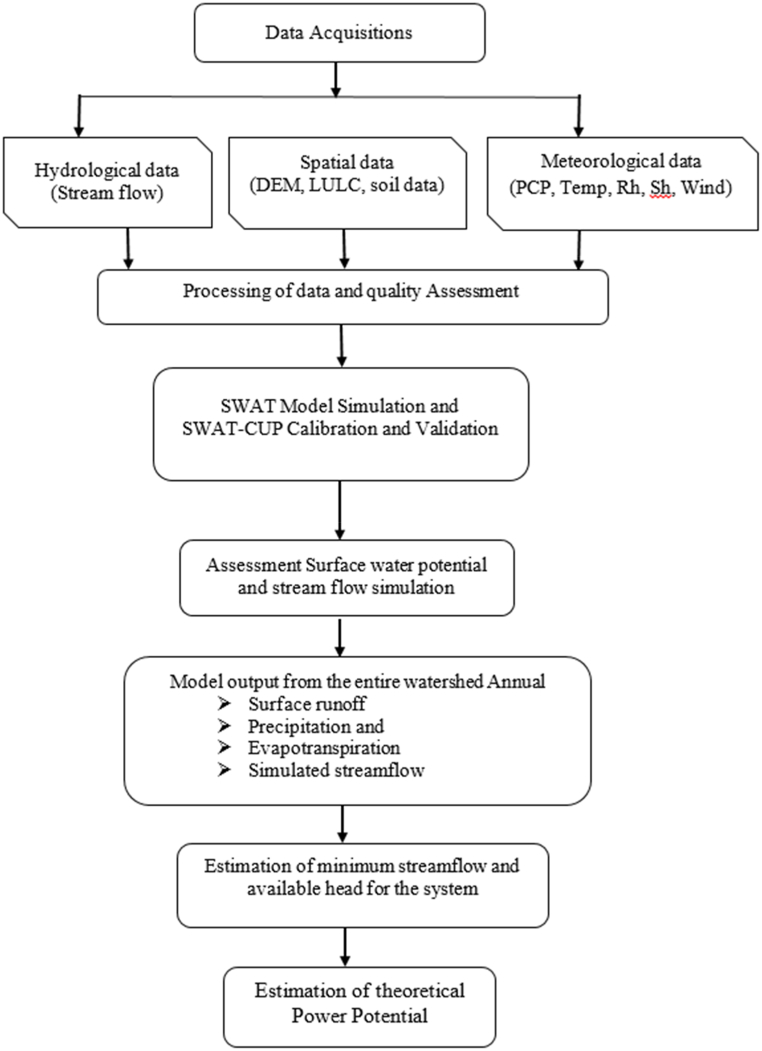
Conceptual frameworks of the study.

The conceptual framework for this study is summarized in Fig. 5.

#### 2.2.1 Data collection

The National Meteorological Agency (NMA) provided the time series daily observed meteorological data for the study, including rainfall, maximum and minimum temperatures, humidity, sunshine hours, and wind speed. The Ministry of Water and Energy's (MoWE) provided the spatial data including DEM, land use, streamflow, land cover, and soil data, which were ensemble together to prepare the ArcGIS maps.

### 2.3 Hydrologic modeling

A hydrological model was used to mimic the complicated reality. This is an ensemble of interdependent or interacting parts that work together to generate a complex system. The primary goal of the hydrologic system analysis was to forecast the system's output by analyzing its operation. The models treat the hydrological cycle as a system that comprises the different components as inputs like precipitation and outputs (runoff), using a set of equations that link the inputs and outputs.

#### 2.3.1 Hydrologic model selection criteria

Numerous criteria like graphical user interface, computer operation system, input/output management, and structure, or users add on expansibility can be used for choosing the right hydrologic model. These criteria are always project-dependent since every project has its own specific requirements and needs. Based on those factors, Arc SWAT model, which is one of the commonly used conceptual models for simulating stream flows and evaluating surface water potential has been chosen to predict the surface water potential in the watershed [27].

#### 2.3.2 SWAT hydrologic model

USDA-ARS, the Agriculture Research Service of the US Department of Agriculture, created the Soil and Water Assessment Tool (SWAT) model. This conceptual model operates on a step-by-step continuous time basis**.** SWAT (Soil and Water Assessment Tool) a theoretical model that operates on a daily time step model followed by a comprehensive, continuous-time, process-based, and semi-distributed conceptual river basin model [28,29] has been adopted in this research work.

### 2.4 Hydrological data

For calibration and validation of the originally simulated flow, regular observational flow data

from the gauged area, near Gubrie station was used from 1990 to 1999 and 2000–2006 respectively. The flow data for the station from the Ministry of Water Resources and Energy (MoWE), is the primary source of hydrologic information (Table 1).

**Table 1:** Summary of the input data.

Data types		Source	Period	Remark
Spatial Data				(DEM Size)
DEM		Alaska site	2019	12.5∗12.5
LULC		MoWE	2007 & 2016	–
Soil		FAO	2012	–

**Hydrologic data**	**Gauge Stations**			

Streamflow	Near Gubrie	MoWE	1992–2006	Daily

**Metrological Data**	**Weather Stations**			

PCP	Welkite, Imdibir & Gunchire Welkite, Imdibir & Gunchire Welkite, Imdibir & Gunchire Welkite, Imdibir & Gunchire Welkite, Imdibir & Gunchire	NMA	1987–2019	Daily
TMP		NMA	1987–2019	Daily
RHMD		NMA	1987–2019	Daily
SLR		NMA	1987–2019	Daily
WND		NMA	1987–2019	Daily

Where; NMA: National Metrological Agency; MoWE: Ministry of Water and Energy; FAO: Food and Agricultural Organization; PCP: Precipitation; TMP: Temperature; RHMD: Relative humidity; SLR: Solar radiation; WNDS: Wind speed; DEM: Digital elevation model; LULC: Land use land cover.

### 2.5 Data processing for the Arc SWAT model

#### 2.5.1 Filling missing rainfall data

Hydrological and metrological data were used due to poor record, lack of continuous follow up and other factors. To fill the missing data, the normal ratio method, arithmetic mean method and inverse distance methods were adopted. These methods also depend on the number of the surrounding index stations or neighboring stations. The inverse distance weighted (IDW) is a scientific analyses method used for index stations up to three or less. In estimating missing rainfall data [30], established that the IDW method is the most suitable method among the arithmetic mean and normal ratio methods for rarely available stations. Mid-country stations use the standard ratio method, whereas up-country stations use the arithmetic mean method (sufficient gauging stations or dense gauging stations). When there are four or more gauging stations, the arithmetic mean and standard ratio methods are used. Since the number of stations available in this study area is only three, the inverse distance weighted (IDW) method is used, which assumes that the precipitation at a given station is influenced by the distance of all stations from it. The weights of each sample's precipitation value are inversely proportional to its distance from the estimated point. The method is adopted by considering the stations with missing rainfall value and is taken as the origin (0,0). For the other neighboring stations and then coordinates of surrounding index stations (x_i_, y_i_) are found. Each index station's weightage (Wi) is equal to the inverse of its separation from the absent station. The IDW method for the missing rainfall data in the station x or Px is given in Equation (1).

(1) $$\text{Px}=\frac{\sum_{i=0}^{n}P_{i}\text{Wi}}{\sum_{i=0}^{n}\text{Wi}}=\frac{P1W1+P2W2+\ldots +\text{PnWn}}{W1+W2+\ldots +\text{Wn}}$$

Where; $\text{Wi}=\frac{1}{{\text{Di}}^{2}}=\frac{1}{{\text{Xi}}^{2}+Yi^{2}}$

Where; Px is the Precipitation of the missing station, P1, P2 … Pn are the Precipitation of index surrounding stations, W1, W2 … Wn are the Weightage of each index station.

#### 2.5.2 Checking rainfall data for consistency

Though it is difficult to carry out direct analysis to detect possible errors in climatic data, there is a probability to check the data consistency of individual stations with some reference stations, since it has a wide range of applications in hydrological fields and is considered accurate. Double mass curve, a graphical technique used, compares a station's record with those of its neighboring stations to determine and correct any inconsistencies. It was possible to distinguish this contradiction from the period when the major shift occurred [31]. A significant change observed in the regime of the curve indicated that rainfall data is inconsistent needs be corrected (Equation (2)).

(2) $$\text{Px}=\text{Po}*\frac{\text{bo}}{\text{ba}}$$

Where; Px is the corrected precipitation at any time, Po is the originally recorded precipitation at the time, bo is the correct (straight line) slope of the double mass curve, and ba is the original slope of the double mass curve.

#### 2.5.3 Determination of areal rainfall

The areal rainfall of the specific catchment stations is determined by the Thiessen polygon method. In this method, weights were assigned to each station according to the catchment area which is closer to that station than to any other station, because different stations give a reasonable amount of weight, the Thiessen-polygon approach of determining the average precipitation across a region was preferable to estimate the arithmetic-average. Furthermore, with a fixed network of stations, the rain gauge stations beyond the catchment region were also easily and successfully employed [32]. For the study area, the available stations around the river are three, but two of them are in polygon and are considered for the estimation of average precipitation for the catchment (Equation (3)).

(3) $$P_{\text{megecha}}=\frac{\left(P_{\text{im}}A_{i}+P_{\text{gu}}A_{\text{gu}}\right)}{\left(A_{\text{im}}+A_{\text{gu}}\right)}$$

Where; P_megecha_ is precipitation on the Megecha watershed, P_im_ and P_gu_ are precipitations of Imdibir and Gunchire stations respectively, A_im_ and A_gu_ are area covered by Imdibir and Gunchire stations.

#### 2.5.4 Filling missing streamflow data

The missing data for streamflow at the selected gauging stations can be filled by using different methods [32]. The linear regression analysis method was used for this study to get the most appropriate result. The method is done by sketching the graph of available streamflow data with the respective periods using the correlation coefficient and linear line equation.

#### 2.5.5 Checking stream flow data consistency

Once the filling of missing data was conducted, the consistency of the recorded flow data was checked by applying outlier tests for higher and lower outlier limits. The outliers were either the data points that deviate considerably from the overall trend or as peaks that deviate significantly from the group's overall trend. Particularly, for small samples, the magnitude of statistical parameters estimated from the data can be strongly impacted by the inclusion or exclusion of these outliers [33]. Procedures for testing outliers required were proper judgment involving both mathematical and hydrologic considerations and detail mathematical explanations (Equation (4)).

(4) $$X_{H}=X_{\text{av}}+K_{N}*S$$

Where; X_H_ = Logarithmic high-outlier test threshold, X_av_ = average value, S = standard deviation.

K is the frequency factor of the test statistic that depends on the significance level α and the sample size n. Equation (5) represents 10 % significance level and sample size n, which is an approximate expression for K.

(5) $$K_{1O\%}=-0.9043+3.345\sqrt{\text{logn}}-0.4046\text{logn}$$

Where, K_N_ = 10 percent significance-level critical value for outlier test statistic for samples of size n from the normal distribution.

If the logarithms of values in a sample are greater than X_H_, it is considered as high outliers. If it indicates a high outlier as a maximum over an extended period, the outlier is treated as historic flood data and excluded from the analysis.

If the record does not contain sufficient information to adjust for high outliers, they should be retained as part of the systematic record. A similar equation can be used to detect low outliers (Equation (6)).

(6) $$X_{L}=\text{Xav}-K_{N}S$$

Where; X_L_ = Logarithmic low-outlier test threshold.

Flood peaks considered as low outliers are deleted from the record to which, a conditional probability was applied. During the outlier test for Megecha river flow at the Gubrie gauging station, the station skewness (G) was found to be 0.139, which is between ± 0.4, so that tests for both high and low outliers were applied and the results were obtained [34]. For 10 % of significance level and sample size n, the following equations can be justified for an approximate expression of K (Equation 7-Equation (12))

(7) $$K_{10\%}=-0.9043+3.345\sqrt{\text{logn}}-0.4046\text{logn},\text{where}\;n=30$$

(8) $$K10\%=-0.9043+3.345\sqrt{\text{logn}}-0.4046\text{logn},\text{where}\;n=17,\text{then}\;K=2.308$$

(9) Highoutliertest,XH=Xav+KNS,=0.659+2.308∗0.118,XH=0.931

(10) $$\text{QH}=10^{0.931}=8.534m^{3}/s$$

(11) Lowoutliertest,XL=Xav−KNS,=0.659−2.308∗0.118,XH=0.388

(12) $$\text{QL}=10^{0.388}=2.442\;m^{3}/s$$

The outlier test result shows that the largest recorded value (7.334 m^3^/s) that does not exceed the high outlier test threshold value (8.534 m^3^/s) and the smallest recorded value (3.152 m^3^/s) is not below the low outlier test threshold value (2.442 m^3^/s). So both high and low outliers were not detected.

### 2.6 SWAT model setup

#### 2.6.1 Watershed delineation

Initiating the research work, watershed delineation was used to divide the watershed unit into different subunits. DEM data was the feeder for the primary input in SWAT model to define the watershed and sub-basins. The heterogeneity of the watershed is described by the division of the watershed into distinct sub-watersheds. A watershed was subdivided into several sub-watersheds in SWAT, which are further subdivided into hydrologic response units (HRUs) with similar land use, management, topographical, and soil characteristics.

#### 2.6.2 Hydrological response unit

HRUs were created by assigning threshold values for land use and land cover, soil, and slope percentage to the sub-watersheds. The watershed's land use, soil, and slope characterization were done with commands from the Arc SWAT Toolbar's HRU analysis menu. These tools made it possible to import raster land use and soil maps into the current project, which was evaluated using slope characteristics and assessing land use/soil/slope class combinations in delineated sub-watersheds. The catchment was divided into fifteen (15) sub-basins and the sub-basins were further divided into sixty-six (66) HRUs.

Slopes of three classes (0–15, 15–30, and 30–9999) were applied and slope grids were reclassified based on the watershed's minimum, maximum, and mean slope estimates. The grids for land use, soil, and slope were then overlaid.

#### 2.6.3 Weather generator

A common problem in Ethiopia is the lack of complete and realistic long-term climatic data. As a result, the weather generator solves this issue by extracting data from observed data. The Arc SWAT modeling required daily metrological data. For the simulation, climate data from January 1st, 1988, to December 31st, 2019, were used. To deal with weather details, it was saved in a special Arc SWAT tabular and supportive file format. They were saved in CSV (delimited comma) excel file format and read by the Arc SWAT. The study area's geographical location and names of weather gauging stations were entered into the Arc SWAT database. The data provided the most accurate precipitation and temperature information. Some meteorological data, such as wind speed, daily sunshine hour, daily wind period, and relative humidity data, are only available at Imdibir station and were created by the model for the other stations. The level of accuracy of climate data available in Imdiabir is checked through Homogeneity, consistency and trend test analysis.

#### 2.6.4 Hydrologic components of Arc SWAT

There are two parts to the hydrological simulations of a watershed. The initial stage of the hydrologic cycle is the land phase, which controls the amount of water, sediment, nutrients, and pesticides that enter the channels of each sub-main basin. In the land phase of the hydrological cycle, hydrological components include canopy storage, infiltration, redistribution, evapotranspiration, lateral subsurface flow, surface runoff, wetlands, tributary channels, and return flow. The movement of water, sediments, nutrients, and organic compounds across the network of channels in the watershed to the outflow known as the routing phase, is the second stage of the hydrological cycle. Using the water balance equation from the land phase of the hydrologic cycle as a basis, Arc SWAT simulates hydrological components [31] (Equation (13)).

(13) $$\text{SWt}=\text{SW}+\sum_{i=1}^{t}\left(\text{Ri}-\text{Qi}-\text{ETi}-\text{Pi}-\text{QRi}\right)$$

Where; R is the daily precipitation in i days (mm), Q is the runoff in i days (mm), and SW is the soil water content. The time in days for the simulation period t is represented by i. P stands for percolation (mm), QR for return flow (mm), and ET is for evapotranspiration (mm).

#### 2.6.5 Surface runoff generation

If the rate of precipitation exceeds the rate of infiltration, surface runoff occurs. For estimating

surface runoff, Arc SWAT provides the (SCS) curve number methods. The number equation for the SCS curve is as follows in Equation (14) [35,36].

(14) $$Q=\frac{{\left(P-\text{Ia}\right)}^{2}}{\left(P-\text{Ia}\right)+S}$$

where Ia is initial abstraction in millimeters, S is theoretical maximum retention, P is effective rainfall in millimeters, and Q is runoff depth in millimeters. However, the function of hypothetical maximum retention S is represented by the first abstraction, Ia. Therefore, where Ia = λS when λ = 0.2 then Ia = 0.2 ∗ S.

Substituting in to Equation (14) it gives Equation (15) [35].

(15) $$Q=\frac{{\left(P-0.2S\right)}^{2}}{\left(P-0.8S\right)}$$

Where; P is more than 0.2∗S, runoff happens. The slope of the watershed, the type of soil, and land use management all affect the potential retention parameter. Equation (16) describes how the dimensionless parameter CN and the probable maximum retention S are connected [20].

(16) $$S=\frac{25400}{\text{CN}}-254$$

#### 2.6.6 Arc SWAT model performance and evaluation

The Arc SWAT model is calibrated and validated monthly to estimate the flow for the Megecha watershed using a time series data set of 17 years (1990–2006). The watershed was subdivided into 15 sub-basins based on a chosen threshold area and a total area of 1176 km^2^. The overlays of land use, soil, and slope maps have resulted in the definition of 66 HRUs.

Observed historical streamflow from Megecha River was used to calibrate and validate the model. These comparisons were carried out considering the statistical parameters mentioned earlier. Model calibration was carried out to evaluate the agreement between simulated and observed stream flows and evaluate the performance of the hydrologic model.

For Megecha River, 10 years of observed historical time series data from 1990-to 1999 was used for calibration, and the data of a period of 7 years or from 2000-to 2006 is used for validation. Validation for this study was used to determine the effectiveness of the calibrated parameters in predicting the flow discharges at the watershed.

Different statistical metrics such as coefficient of determination (R^2^), Nash-Sutcliffe modeling efficiency (NSE), and percent bias (PBIAS) were used to assess the overall performance of the Arc SWAT model's calibration and validation.

i. Coefficient of determination (R^2^): The index of similarity between calculated and simulated values is known as the Coefficient of Determination. R^2^ always has a value between 0 and 1. The higher the R^2^ value reaches 1, the better the model's performance, while R^2^ values less than 0.6 imply low model performance. According to Ref. [30], flow simulation is judged as satisfactory if R^2^ ≥ 0.6 and it can be estimated by using Equation (17).

(17) $$R^{2}=\frac{\sum \left(\text{Qo}-\bar{\text{Qo}}\right)*\left(\text{Qs}-\bar{\text{Qs}}\right)}{{\left(\sum_{i=1}^{n}{\left(\text{Qo}-\bar{\text{Qo}}\right)}^{2}*\left(\sum_{i=1}^{n}{\left(\text{Qs}-\bar{\text{Qs}}\right)}^{2}\right)\right)}^{0.5}}$$

ii Nash-Sutcliffe Efficiency (NSE): The Nash-Sutcliffe efficiency is a normalized statistic that calculates the magnitude of residual variance versus calculated data variance. The letter NS denotes how well the observed versus simulated data plot matches the 1:1 axis. It has a range of values from -ve to 1, with 1 indicating complete harmony between simulated and observed variables. The formula for NSE is presented in Equation (18) [22].

(18) $$\text{NSE}=1-\frac{\sum {\left(\text{Qo}-\text{Qs}\right)}^{2}}{\sum {\left(\text{Qo}-\bar{\text{Qo}}\right)}^{2}}$$

iii Percent bias (PBIAS): represents the average propensity of the simulated data to be greater or smaller than their observed equivalents. PBIAS should ideally be at 0.0, with low magnification values denoting precise model simulation. Model underestimation bias is shown by positive values, and model overestimation bias is indicated by negative values as shown in Equation (19) [7].

(19) $$\text{PBIAS}=\left[\;\frac{\sum_{i=1}^{n}\left(Q_{i}^{\text{obs}}-Q_{i}^{\text{sim}}\right)*\left(100\right)}{\sum_{i=1}^{n}\left(Q_{i}^{\text{obs}}\right)}\right]$$

Where; Q^obs^ is observed stream flow, Q^sim^ is simulated Stream flow, and n is the total number of observations.

#### 2.6.7 Model sensitivity analysis, calibration, and validation

##### 2.6.7.1 Sensitivity analysis

A complex hydrologic model is characterized by many variables. As a result, models must be conditioned to balance simulated and measured data to achieve a good fit. The calibration of the model can be done manually or automatically. In all cases, it's a good idea to use methods like sensitivity analysis to back you up. A parameter sensitivity analysis reveals the parameters are most responsible for the output variance caused by input variability [34].

Calibration for a limited number of influential parameters can be performed based on this knowledge. As a result, before the calibration phase, a sensitivity analysis was performed to define important parameters for model calibration in this report. The sensitivity of the stream flow was calculated using the watershed's average monthly streamflow data at gauging stations near Gubrie.

##### 2.6.7.2 Calibration

Model calibration was carried out in response to the sensitivity analysis results to achieve the best values for sensitive parameters. Arc SWAT offers three calibration methods; auto-calibration, manual calibration, and a hybrid of the two. SUFI-2 with an uncertainty analysis model was used to run the auto-calibration method in SWAT-CUP on monthly time steps using the watershed's average calculated streamflow data. The model was run using the best parameter output values after the auto-calibration run was completed, and the simulations were compared to observed streamflow data [27].

The base flow adjustment came after the surface runoff adjustment. When adjusting the most sensitive factors influencing the base flow, the same methodology was used as before. Following each calibration, the Nash and Sutcliffe simulation efficiency (NSE) as well as the regression coefficient (R^2^) were examined. Developers of Arc SWAT believed that hydrology could be calibrated to an acceptable level at R2 > 0.6 and NSE >0.5; these values were also taken into consideration as references in this investigation. The model flow calibration period (1990–1999) and the parameters of the available model input data are used in this investigation.

##### 2.6.7.3 Validation

To compare the model outputs with an independent data set without altering the parameter values, validation was also done. An unadjusted comparison of the model outputs with an independent data collection is called application validation. To make sure the simulated values were within the precise bounds; the three model output values were also evaluated during this step. The watershed's seven years of observed streamflow data, from January 1, 2000, to December 31, 2006, were utilized in the validation process. To confirm that the simulated values remain within the accuracy constraints, the statistical criteria (R^2^, NSE, and PBIAS) that were employed throughout the calibration method were also examined here. PBIAS < ± 15, R^2^ > 0.6, and NSE >0.5.

### 2.7 Hydrologic analysis

Hydrologic evaluations were used to provide value/values of stream discharges that were used in selecting the size of the units and to determine annual energy production. This implies that site-specific hydrologic data were needed to give the time variation of stream discharge. Rarely is a stream gauge located at the desired hydropower site. Different techniques like hydrologic models can be used to simulate stream flows for ungauged sites, one of these models is SWAT (Water and Soil Assessment Tools) model.

#### 2.7.1 Simulation of streamflow for an ungauged catchment or watershed

One of the key factors in estimating hydropower, hydrologically analyzing catchments, and analyzing river behavior is streamflow [37,38]. This variable was important for the monitoring and management of various activities at the catchment scale, including the design of water infrastructure, irrigation schedule, study of river behavior, flood warning and control, and environmental management. It also plays a crucial role in the quantity and quality of water resources [33]. The Arc SWAT hydrologic model is used to simulate streamflow for ungauged catchments or sub-watersheds by using the sensitive parameters of the watershed by applying manual calibration. This can generate and predict streamflow for ungauged identified sites in the watershed. The streamflow for the identified and ungauged sites were simulated by using manual calibration in the Arc SWAT model.

#### 2.7.2 Estimation of minimum available flow

To estimate the potential energy that might be developed at a specific site, it is convenient to use the minimum average flow value for the year as the flow value in the power equation. Using an average annual flow value and an average value for the topographic head in the power equation and multiplying the results by the total number of hours in a year gives the total annual energy available in Kilowatts hours, KWHs. A flow-duration curve is a commonly used tool to describe the minimal flow available at a place. An illustration of the average time availability of flow is a flow duration curve. Plotting flow against the percentage of time when flow is equal to or greater than is represented by the curve. The curve is computed from a sequential list of flows that are representative of flows available for power production at a particular point of interest in a stream. The curve is a very useful tool in hydrologic analysis in general and especially useful for hydropower studies. In hydropower analyses, the flow-duration curve can be used to determine estimated power and energy from a proposed hydropower installation. The flow-duration curve provides information on low flows which are necessary to design storage capacity and/or select appropriate turbines. Therefore, a careful determination of flow duration curves is an essential part of the hydropower system development.

#### 2.7.3 Computation of flow duration curve

For this research, the ranked flow technique is used as it gives more correct results than the second method which averages out extreme events. The percentage of exceedance is computed by using Equation (20) [39].

(20) $$\%\;\text{of}\;\text{Exceedance}=\frac{m}{n+1}*100$$

Where; m is the rank of ordered flow, and n is the total no. of sample.

#### 2.7.4 Development of flow duration curves

##### 2.7.4.1 Length of flow records

The development of the flow duration curve depends on the length of the recorded flow. However, for many sites, considerably less than 30 years are available. Most of the gauging stations on small rivers have got historical data less than the minimum required i.e., 30 years. In addition to this long time and recent flow data records are essential to estimate the current power potential of any streamflow. In order to lengthen a period of record and provide flow data for ungauged sites or regions, hydrological models such as an Arc SWAT model simulation or correlation and regression are typically utilized for sites with shorter records in order to estimate the reliable quantity of streamflow. Flow simulation using the Arc SWAT modeling method was used to extend the flow record periods from short time records to a long time and to generate flows for ungauged areas [40].

##### 2.7.4.2 Flow-duration curves for different sites

A flow-duration curve was plotted in different sites based on available flow data. However, observed flows for different sites may not be available simply. In this case, different mechanisms can be applied to extend or generate stream flows for ungauged areas, like flow transfer and flow simulation methods. Flow simulation by means of Arc SWAT model watershed simulation is applied to generate flows for the ungauged years and ungauged areas.

##### 2.7.4.3 Estimation of available head

The head required for hydropower development is computed from the water level elevation difference between the proposed site and the powerhouse station, or it is the height drop from intake location to turbine location [41]. It depends on the topographic slope characteristics of the study area, and the amount of minimum streamflow, and this head is also filtered by the minimum head which is basically defined by the type of turbine. The Low- Head turbine deal with a head in the range between 2 m and 35 m. The available head or height drop of water in hydropower sites can be estimated by physical site investigation or from the DEM map of the study area by the GIS-based method. GIS-based method is used. Hence the gross head can be estimated by taking the elevation differences, it is the vertical height through which the water drops and is measured in meters or feet.

#### 2.7.5 Estimation of the hydropower potential of the steam flow

After estimating the minimum streamflow of the river and the available head of the system the next task is to estimate the theoretical power potential of the stream for the selected suitable site. The theoretical hydropower potential of the river flow can be estimated by using the minimum available flow of the river and the available head drop or elevation difference between the proposed site and turbine location as identified above. It is the product of the minimum available streamflow, the height drops, and the specific weight of the falling water to the turbine. The minimum flow of the river is the flow that is available throughout the year including the dry season and can be obtained from the flow duration curve by using the percent of exceedance value from 80 to 95 % [3,16,42,43]. The available head is the head or the vertical elevation between the initial intake location point and the proposed turbine location area. Thus, multiplying these two main parameters by the specific weight of water and the turbine efficiency; the gross power potential of the river can be estimated in Equation (21) [42].

(21) $$P=\eta *\;\gamma_{w}\;*H_{\text{net}}*Q$$

Where P stands for power potential (KW), η for hydraulic turbine efficiency (%), γw for specific weight of water (9.81 kN/m^3^), H for net head of flow (m), and Q for river flow rate (m^3^/s). Small hydropower systems' power output is often determined using a firm discharge that ranges from 80 % to 95 % of likelihood of exceedance (Q80 and Q95) [38]. In light of this, the analysis employed simulated stream flow from the chosen site that corresponded to a 90 % likelihood of exceedance (Q90).

## 3 Results and discussions

### 3.1 Watershed delineation

#### 3.1.1 Basin and sub-basin boundaries

**Fig. 6:**
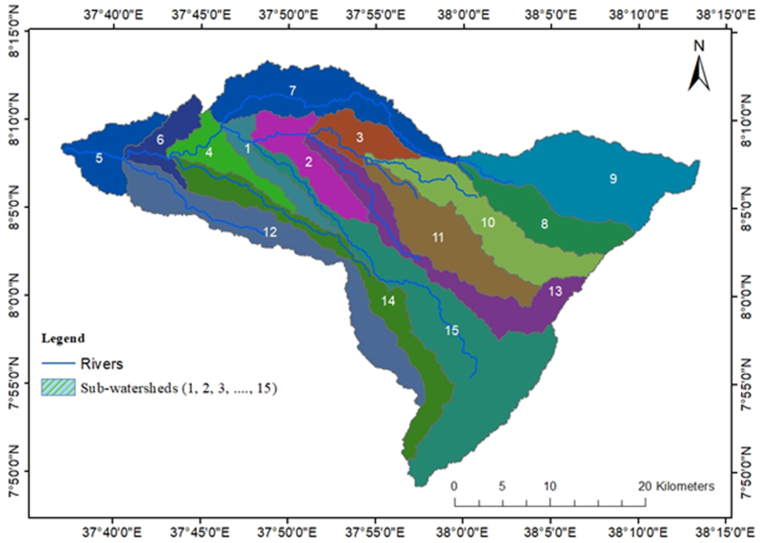
Sub-watersheds in the Megecha watershed.

Basin and sub-basin boundaries have been delineated using the topographic maps, SRTM DEM (12.5m∗12.5m resolution). As the study area is not big it is divided into small sub-basins (sub-watersheds) based on the drainage pattern and the modeling requirements. Sub-basin boundaries are extracted through the automatic process using the programs available in GIS software. Flow accumulation grids were computed for sub-watershed delineation, keeping given the drainage pattern. Megecha watershed has been subdivided into 15 sub-basins. Fig. 6 shows sub-basins of the Megecha watershed.

#### 3.1.2 Sensitivity analyses of the model

Sensitivity analysis was carried out to identify which model parameter is most important or sensitive. After the pre-processing of the data and Arc SWAT model set up, simulation was done for the period 1988 to 2006 and the simulation for the period January 1, 1988, to December 31, 1989, was considered a two-year warm-up period. Warm-up periods are periods used by the Arc SWAT simulation process as sample simulation periods, and they are base periods to simulate the rest period of the years.

The period from 1990 to 2006 was used for sensitivity analysis. In this study, for the purpose of model calibration, 20 parameters were identified as sensitive parameters with different degrees of sensitivity, and which are selected based on the dominant features of the watershed characteristics.

The most sensitive parameters, their ranking, and description are shown in Table 2.

**Table 2:** The most sensitive parameters, their rankings, and descriptions.

S.No	Parameter Name	Min value	Max value	Fitted value	t-Stat	p- value	Rank
1	CN2.mgt	35	98	78.470	−13.715	0	1
2	SOL_K(.).sol	0	2000	113.333	−12.694	0	2
3	SOL_BD(.).sol	0.9	2.5	1.033	−10.350	0	3
4	SOL_AWC(.).sol	0	1	0.430	9.155	0	4
5	SLSUBBSN.hru	10	150	108.467	7.664	0	5
6	CANMX.hru	0	10	2.189	3.584	0.001	6
7	REVAPMN.gw	0	500	265.00	−2.110	0.035	7
8	ESCO.bsn	0	1	0.428	1.123	0.262	8
9	EPCO.bsn	0	1	0.857	1.004	0.316	9

Where; CN2.mgt: Soil conservation service (SCS) runoff curve number.

SOL_K (…).sol: Saturated hydraulic conductivity.

SOL_BD (…).sol: Moist bulk density.

SOL_AWC (…).sol: Available water capacity of the soil layer.

SLSUBBSN.hru: Average slope length.

CANMX.hru: Maximum canopy storage.

REVAPMN.gw: Threshold depth of water in the shallow aquifer for “revap” to occur (mm).

ESCO.bsn Soil evaporation compensation factor.

EPCO.bsn: Plant uptake compensation factor.

A description of these parameters with their effect is provided by both Arc SWAT and the SWAT-CUP User's manuals. Predicted flow was found to be most sensitive for soil and land use properties: curve number (CN2), saturated hydraulic conductivity (SOL_K), moist bulk density (SOL_BD), and available water capacity of the soil layer SOL_AWC were among the most sensitive parameters.

#### 3.1.3 Model calibration and validation

The model was calibrated for the sub-basin 7 watershed, equipped with an automatic runoff recorder near the Gubrie gauge station. In this study, model calibration was conducted using the observed and model-simulated flow using the Arc SWAT model. Several simulations have been run and applied to achieve the best model efficiency between the observed and simulated flows. The goodness of fit and efficiency of the model were used to assess the model performance. The performance of the model was demonstrated by the correlation coefficient (R^2^) and the Nash-Sutcliffe simulation efficiency (NSE) values. According to Ref. [30], for the model to be accepted or satisfactory for model prediction, the model performance statistics should be R^2^ > 0.6 and NSE >0.5 for daily and monthly simulated values. The flow data from 1990 to 1999 was used for calibration purposes and the evaluated values of the R^2^ = 0.84 and NS_E_ = 0.81, and PBIAS is −4.8 % which are within the acceptable limits.

**Fig. 7:**
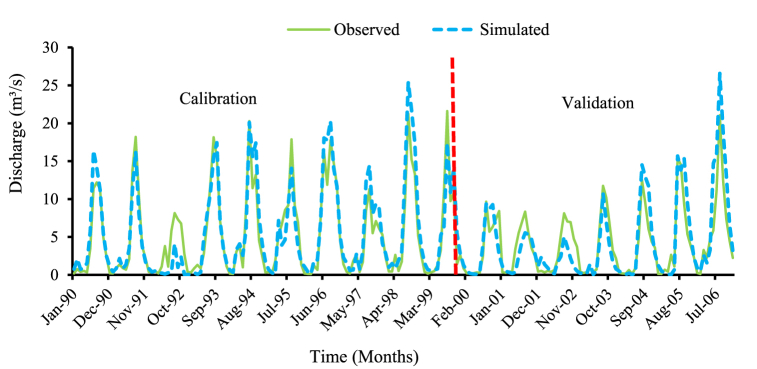
Flow calibration and validation of monthly observed vs. simulated flows.

After the calibration process, the model was validated with calibrated parameters by using an independent set of observed flow data which were not used during model calibration. Here the model validation was done using independent data sets of seven (7) years from Jan 1, 2000–Dec 31, 2006, without further adjustment of the parameters of flows. Good agreement between monthly observed and simulated flows was obtained as demonstrated by the observed and simulated flows. The performance of the model was demonstrated by the correlation coefficient (R^2^) of 0.78, and Nash-Sutcliffe model efficiency (NSE) of 0.72, with a percent of bias PBIAS -4.6 %. The statistical parameters again suggested that the model was also performed well in the validation period for R^2^ > 0.6, NSE >0.5 and PBIAS < ± 15 % [44]. Fig. 7 shows the calibration and validation of the model in the watershed for observed periods and overall Summary of model evaluation estimated numerical values are shown in Table 3.

**Table 3:** Summary of model evaluation estimated numerical values.

Model runs	Model performance evaluation parameters			Remarks
	R^2^	NSE	PBIAS (%)	
Calibration	0.84	0.78	−4.6	OK!
Validation	0.81	0.72	−4.8	OK!
Ranges	>0.6	>0.5	±15 %	Acceptable

These values are considered adequate statistical values for acceptable model calibration. Hence, the performance of the calibration is based on R^2^ and NSE between the simulated and measured daily and monthly discharge as well as a visual comparison of the simulated and measured streamflow hydrographs.

### 3.2 Surface runoff potential of the watershed

After the successful calibration process and obtaining the sensitive parameters of the watershed, the model simulation was run again on a daily time scale to simulate the monthly stream flows for the identified sub-watersheds and to show the runoff from each HRU (SWAT predicts runoff from HRU). Analysis of the results was done in the context of the sub-watersheds.

**Fig. 8:**
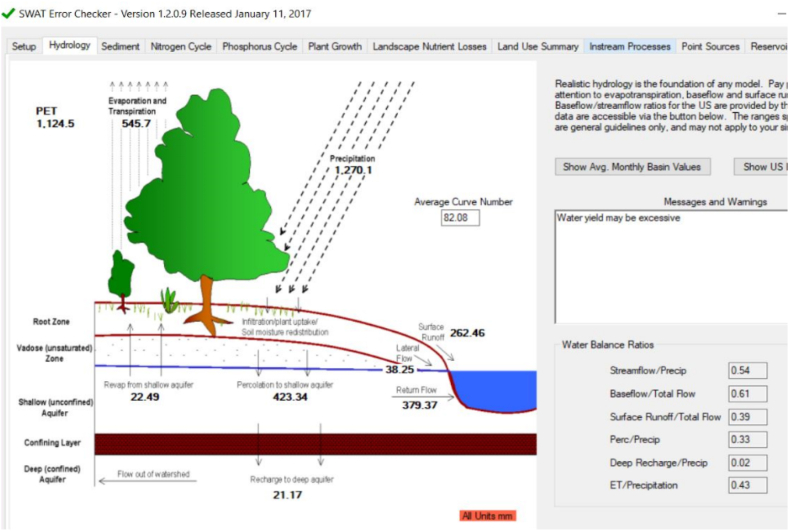
The hydrologic cycle of the Megecha watershed (1988–2019).

Arc SWAT modeled runoff according to the land use, soil type, and slope since land-use types vary with the soil type. Thereby, 66 HRUs were created for the sub-watershed according to the given land uses in the sub-watershed and soil types of the watershed area. The Arc SWAT model run results for the Megecha watershed showed that the watershed receives 1270.1 mm of mean annual rainfall and/or 105.84 mm of mean monthly rainfall, which is 1493.64MMC and 124.47MMC of rainfall respectively. Evaporation loss takes a significant number in the watershed because the watershed faces high temperature, is covered by green, and wind speed is high which increases the evaporation loss. Hence evaporation loss is found to be 545.7 mm or 43 % of mean annual rainfall. The total annual surface runoff output in the basin is 262.46 mm or 308.65MCM (million cubic meters). This shows that the mean annual surface runoff outflow that the Megecha watershed contributes to the Omo gibe basin is 308.65 MMC which is 20.66 % of the annual rainfall (Fig. 8).

### 3.3 Simulation of stream flows for the identified sites

#### 3.3.1 Mean monthly stream flows

**Fig. 9:**
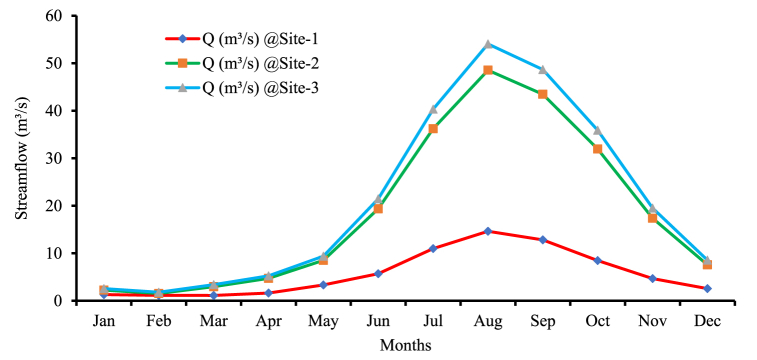
Simulated mean monthly flow at identified sites in the Megecha watershed.

The Megecha watershed has been assessed and the stream flows are simulated by using the Arc SWAT hydrologic model with the sensitive parameters in the watershed after the calibration processes. The stream flow simulation showed that the mean monthly stream flows at the identified sites are varied from a minimum of about 1.130 m^3^/s (February), at site-1 (near the gauged station) and to a maximum of about 54.054 m^3^/s (August), in the last site-3 as shown in Fig. 9.

#### 3.3.2 Mean monthly stream flows comparisons

**Fig. 10:**
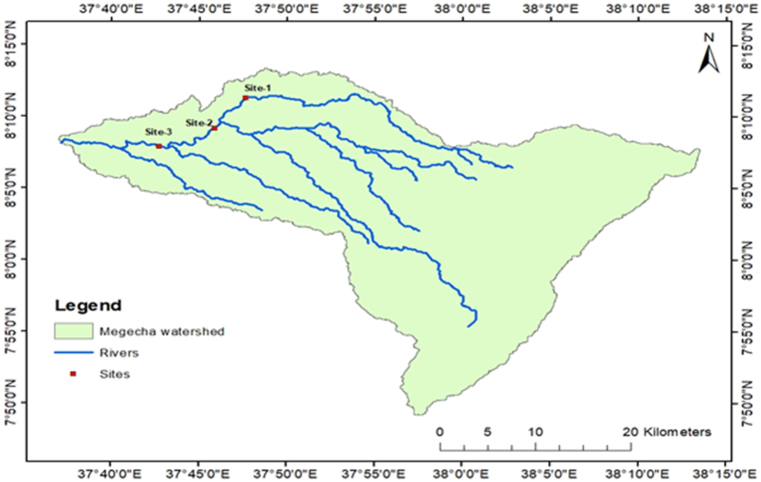
The locations of identified potential sites for streamflow computation.

The mean monthly streamflow of the identified sites is generated using the historical meteorological data of 1988–2019 and the Arc SWAT model. According to the simulated streamflow results in the identified sites, the mean monthly flows range from the minimum of 1.130 m^3^/s to 14.636 m^3^/s, at the gauged station; 1.543 m^3^/s to 48.529 m^3^/s at site-1; and 1.806 m^3^/s to 54.054 m^3^/s at site-2. This shows that from the identified sites, site-2 and 3 have better flows than the other (Fig. 10).

#### 3.3.3 Estimation of minimum available flow

The minimum available flows of the river at the identified sites are estimated from the 30 years 1990–2019 periods simulated flows by using the flow duration curve. The mean monthly flow of each site was arranged in descending order, and the percentage of exceedance from the rank assigned to each value in the order was computed to create the flow duration curve of the detected locations. The hydropower potential of the listed sites was calculated using Q90, the streamflow of the sites that are available for ninety percent of the year.

#### 3.3.4 Developing the flow duration curves for identified sites

**Fig. 11:**
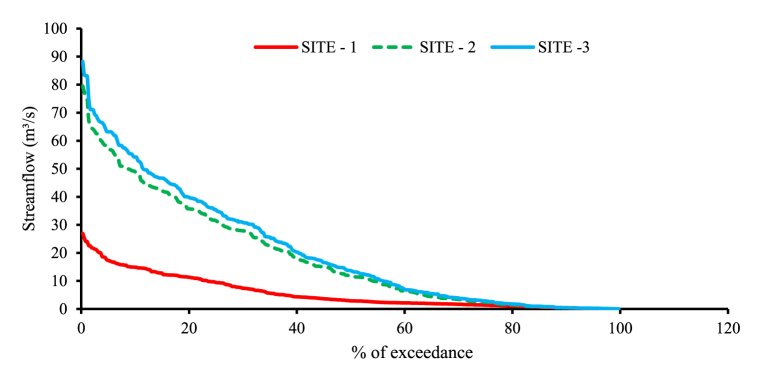
The flow duration curves of Megecha River at the three sites (1990–2019).

The flow duration curves are plotted for all three sites, and the dependable flows for the ninety percent of exceedance (Q_90_) are computed for each site. Fig. 11 shows the flow duration curves of the river at identified sites.

Based on the FDC above the selected minimum flow of the river for the percent of exceedance 90 % (Q_90_) for site 1, site 2, and site 2 are 0.37 m^3^/s, 0.45 m^3^/s, and 0.48 m^3^/s respectively, and these flows are selected to estimate the power potential of the river on each site and this result is comparable with a study by Refs. [12,45].

#### 3.3.5 Estimation of gross heads

**Fig. 12:**
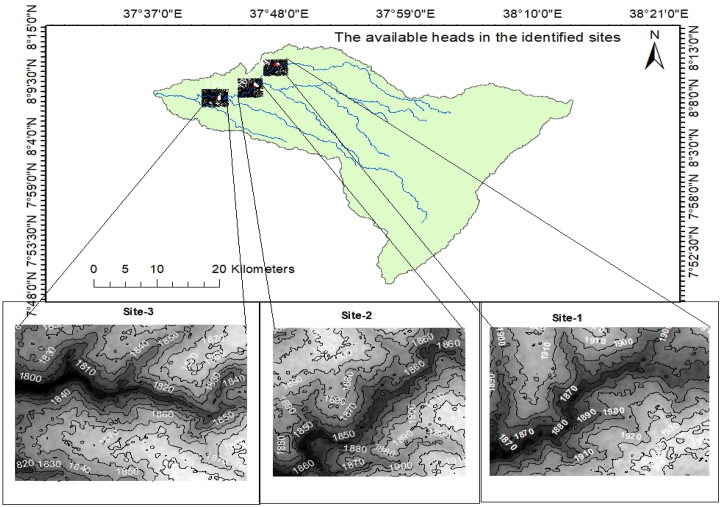
The identified sites for gross head to estimate the power potentials the river.

The available head or height drop of the water used to generate the power is said to be gross head and it is computed from the DEM map of the study area by a GIS-based method (Fig. 12). At the identified site-1 (near to gauge station) the height drops from the maximum elevation at the river reach about 1880m (m.a.s.l) to the minimum 1865m (m.a.s.l); at site-2 the height drops from 1870m to 1845m (m.a.s.l) and at site-3 it ranges from the maximum of 1830m to the minimum of 1810m (m.a.s.l). Accordingly, the available heads at the identified sites are 15, 25, and 20 m at site-1, site-2, and site-3 respectively.

#### 3.3.6 Estimation of the hydropower potential of the river

River water flow Q, the total net head Hn between the turbine's intake and output sections, and the turbine efficiency η all have an impact on the hydropower source's potential. A rainfall-runoff model is used to evaluate Q, and the DEM is used to estimate the drop head as the height difference along the riverbed, ignoring the energy losses along the penstock [10,46,47]. The turbine efficiency is contingent upon both the type of turbine placed in the hydropower plant and the turbine's characteristic curve for a particular supply condition (Q and Hn) (Equation (22)). There are several varieties of turbines, and choosing the best one for a given situation is crucial to the design process.

(22) $$P=\eta \gamma QH_{n}$$

Where; P is installed power capacity (kW), η is turbine efficiency (%), γ is specific weight of water (KN/m^3^), Q is minimum flow (m^3^/s), and Hn is the net head (m).

In this study, hydropower potential of the river was estimated as the gross power using the gross head of the plant to obtain the theoretical power potential of the river. Based on the estimated minimum flows of the river and the computed available gross heads at each of the identified sites (using the DEM map), the theoretical power potentials of the river were estimated as 54.45 KW, 110.36 KW, and 94.18 KW at site-1, site-2, and Site-3 respectively and similar work is conducted [3,46].

### 3.4 Site selection criteria to select the appropriate potential site

The water potential, available head and site suitability is the prominent parameters to select appropriate site for hydropower development [19,48]. So, in this study, the hydropower plant site location is evaluated by considering the essential criteria in addition to the above dependent variables to optimize the power potential of the plants by applying the multi-criteria decision making (MCDM) analysis methods. Multi criteria analysis helps to identify the suitable sites through a decision-making process [45].

#### 3.4.1 Multi-criteria decision making (MCDM) method

This is a method of using multiples variable to exactly identify the lucrative and feasible sites for hydropower generation and detail explanation is available in Ref. [22].

#### 3.4.2 Selection of decision-making criteria

The most important step in a multicriteria analysis is choosing criteria that consider social, environmental, and economic factors [49]. One of the most important criteria applied in this study is the technical criteria which include the annual discharge; gross head, installed capacity, and length of transmission line are analyzed by means of multi-criteria decision analysis.

**Annual discharge (minimum flow):** Fundamental metric was used to determine the energy generation from water. The minimum flow of the plant was computed from the minimum flow of the river using the flow duration curve and was computed for the three identified alternative sites as 0.37, 0.45, and 0.48 in m^3^/s at sites 1, 2, and 3 respectively.

**Gross heads (m):** The height drop elevation of the water from the location of the intake to the powerhouse was assessed. It is an additional fundamental factor for determining hydropower energy generation from water. The gross head of the plant was computed from the topographic DEM map for the identified sites (alternatives) as 15, 25, and 20 m.

**Power potential (kW):** It was estimated and function of the available minimum flow and head of the plant was computed (Equation (16)).

**Transmission lines and its measurement(km):** According to the estimations from the GIS DEM map in addition to the information, the location distance of the society from site-1 was measured as 9 kms, whereas, from site-2 is 8 kms, and from site-3 is about 12 kms. These decision-making criteria are also categorized into beneficial (maximizing criteria); minimum flow, gross head, and power potential, and unbeneficial (minimizing criteria); length of transmission lines.

#### 3.4.3 Evaluation of multi-criteria decision methods

Numerous techniques may be used to assess multi-criteria decision processes, but the most popular techniques are the matrix approach and the analytical hierarchy process (AHP). In this study, the optimal alternative prospective location for the installation plant was ranked and was chosen using the MCDM analytical hierarchy process (AHP) via matrix technique. The selected criteria were arranged, evaluated, and ranked based on the results of each parameter estimated earlier (Table 4, Table 5, Table 6).

**Table 4:**
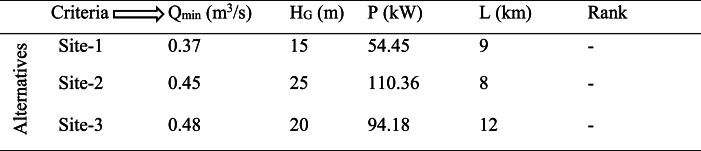
Step 1: Arranging the decision-making parameters (criteria) in the matrix tabular form.

Where; $\underset{\text{Beneficial}\;\left(\text{maximizing}\right)\;\text{criteria}}{\underbrace{Q_{\min}{=\min\;\text{flow},H}_{G}=\text{gross}\;\text{head},P=\text{power}\;\text{potential}}}$, $\underset{\text{Unbeneficial}\;\left(\text{minimizing}\right)\;\text{criteria}}{\underbrace{L=\text{length}\;\text{of}\;\text{transmission}\;\text{line}}}$

**Table 5:**
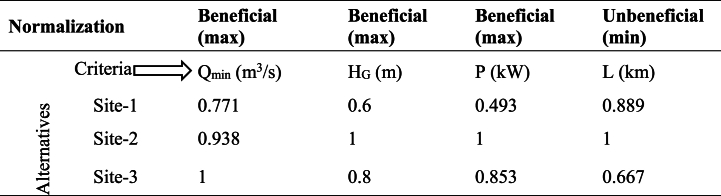
Step 2: Normalizing the criteria for each alternative.

(Normalized matrix).

Normalization: For beneficial: Xij = Xij/maxXij, 0.771 = 0.37/0.48, …

For unbeneficial: Xij = min(Xij)/Xij: 0.889 = 8/9, …

**Table 6:**
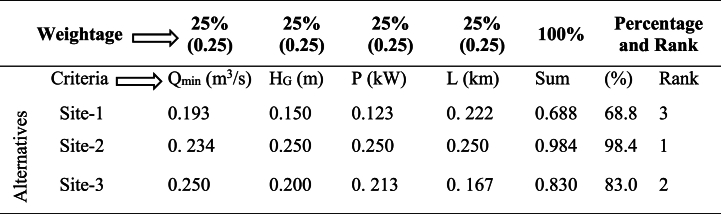
Step 3: Assigning weightage for each parameter (criteria) and compute out of 100 % and rank them.

Where; 0.193 = 0.25∗0.771 … Weightage of each criterion out of 100 %.

According to the evaluations of the decision making criteria for each parameter, the rank of the criteria shows that the best alternative site to select is at site number 2, which has the best alternatives rather than other identified sites. This selected site of Megecha watershed has the power potential to generate about 110.36 kW, which is categorized under small-scale power potentials, and this is a comparable result with the study by Ref. [50].

## 4 Conclusion

The water potential of the watershed was assessed to select potential sites for hydropower by integrating the computational methods with available data. And from the sensitivity analysis, calibration, and validation for the model output have been done successfully by using historical observed streamflow data with the help of SWAT-CUP calibration process. The results of sensitivity analysis showed that CN2, SOL_K, and SOL_BD were the most sensitive parameters than others in the watershed which depend on soil properties and land use, different types of moisture conditions, and the retention parameter (soil water content and potential) of the watershed. The calibration and validation results of SWAT were evaluated by statistical parameters like R^2^, NSE, and PBIAS, and showed a good model efficiency. The mean monthly streamflow of the watershed was simulated successfully for the identified sites with the help of manual calibration in the Arc SWAT model using sensitive parameters of the watershed, and the minimum flow of the river which is used to estimate the power potential at the selected site is also computed by using the flow duration curve and taking 90 % of exceedance is 0.45 m^3^/s. The gross head (25m) was the other variable to generate the power at the selected site and was computed by using the topographic map or DEM-map-based method. Accordingly, the power potential of the Megecha River at the selected site was estimated as 110.36 KW. This power is sufficient to produce power from water for rural electrification. Cross verification with field survey is very important but due to limited budget we can't conduct detail survey of full river flow but instead simple survey for contour map verification is conducted, and further research must include such consideration for detail investigation of river flow. The data collection system and good watershed management practice must be updated for accurate record of hydrological and metrological data. The multi-criteria decision-making analysis was applied to select the best alternative site from the identified alternatives by taking the power estimation parameters as the decision-making criteria. According to the rank of the criteria the best selected alternative site is at site number 2 with optimal result. This shows that the river has the power potential to generate small-scale power at the selected site.

## CRediT authorship contribution statement

**Habtamu Wimego Anore:** Writing – original draft, Formal analysis, Data curation, Conceptualization. **Tarun Kumar Lohani:** Writing – review & editing, Supervision, Methodology, Formal analysis, Conceptualization. **Abebe Temesgen Ayalew:** Validation, Software, Resources, Project administration.

## Data availability

Data will be made available on request.

## Funding

This research did not receive any specific grant from funding agencies in the public, commercial or not-for-profit sectors.

## Declaration of competing interest

The authors declare that they have no known competing financial interests or personal relationships that could have appeared to influence the work reported in this paper.
